# Sensitivity of Commercially Available Influenza Rapid Diagnostic Tests in the 2018–2019 Influenza Season

**DOI:** 10.3389/fmicb.2019.02342

**Published:** 2019-10-11

**Authors:** Yuko Sakai-Tagawa, Seiya Yamayoshi, Yoshihiro Kawaoka

**Affiliations:** ^1^Division of Virology, Department of Microbiology and Immunology, Institute of Medical Science, The University of Tokyo, Tokyo, Japan; ^2^Department of Pathobiological Sciences, School of Veterinary Medicine, University of Wisconsin–Madison, Madison, WI, United States; ^3^Department of Special Pathogens, International Research Center for Infectious Diseases, Institute of Medical Science, The University of Tokyo, Tokyo, Japan

**Keywords:** influenza, rapid diagnosis test, point-of-care testing, sensitivity, specificity

## Abstract

Epidemics of seasonal influenza caused by H1N1pdm09, H3N2, and type B viruses occur throughout the world. Sporadic human H5 and H7N9 virus infections are also reported in particular regions. To treat influenza patients effectively with antivirals, sensitive and broad-reactive influenza rapid diagnostic tests (IRDTs) are required. Here, we tested the sensitivity of 23 IRDTs during the 2018–2019 influenza season for their ability to detect H1N1pdm09, H3N2, H5N1, H5N6, H7N9, and Victoria- and Yamagata-lineage type B viruses. All IRDTs detected all influenza A and B viruses tested but with different sensitivities. Several IRDTs detected the H5 and H7 viruses and the seasonal viruses with similar sensitivity. Such IRDTs might be useful for diagnosing patients infected with H5 and H7 viruses.

## Introduction

Influenza virus infection is one of the most prevalent infectious diseases. Seasonal influenza viruses, including H1N1pdm09, H3N2, and type B viruses, cause annual epidemics, despite the use of vaccines against influenza virus. Other subtypes of influenza A virus from other animal species such as chickens and pigs have caused sporadic human cases. For example, highly pathogenic influenza virus subtype H5N1 is circulating among poultry in Asian countries and Egypt and has transmitted to humans (Harfoot and Webby, 2017). Reassortant H5Nx viruses such as H5N2, H5N6, and H5N8 viruses that possess the hemagglutinin (HA) segment of a highly pathogenic H5N1 virus and the neuraminidase (NA) segment of another subtype have emerged and spread in European and North American countries (Nunez and Ross, 2019). H5N6 viruses sporadically infect humans (Yang et al., 2015), and H5N2 viruses replicate well in mammalian hosts (Pulit-Penaloza et al., 2015; Kaplan et al., 2016). Since 2013, human infections with the avian influenza H7N9 virus have been reported (Gao et al., 2013). During the 2016–2017 season, highly pathogenic H7N9 viruses possessing HA with multi-basic amino acids at the cleavage site were isolated from avian and human cases (Zhang et al., 2017; Zhu et al., 2017). It is difficult to prepare vaccines against these H5 and H7 viruses in a timely manner; therefore, the first line of defense against such viruses is antiviral drugs, such as NA inhibitors and an endonuclease inhibitor.

For antiviral drugs against influenza to be effective, it is important to identify patients infected with influenza virus as soon as possible. In particular, NA inhibitors (oseltamivir, zanamivir, peramivir, and laninamivir) should be administered within 2 days of symptom onset for optimal efficacy (McNicholl and McNicholl, 2001; Yamashita, 2010). Healthcare providers need a rapid, easy, and sensitive diagnosis test for such purposes. Although basic virologic approaches such as virus isolation and RT-qPCR have been used, they are not suitable in the clinical setting because of time constraints and the need for specialized equipment and technique. Influenza rapid diagnostic tests (IRDTs) are now widely used even at the local, small clinic level in Japan. However, conventional IRDTs that are read by the human eye fail to detect influenza viruses at early timepoints after onset (Watanabe et al., 2009, 2011). To improve this situation, some manufacturers have developed IRDTs that are assessed by analyzers. Here, we examined the sensitivity of 23 IRDTs (5 IRDTs that use analyzers and 18 that are assessed by the human eye) that are available in 2018–2019 to detect various isolates of seasonal influenza A and B viruses as well as human and avian H5Nx and H7N9 viruses, which possess the potential to transmit to humans (Richard and Fouchier, 2016).

## Materials and Methods

### Diagnostic Tests

The 23 IRDTs listed in Table 1 were evaluated for reactivity and sensitivity according to the manufacturers’ procedures. The lysis buffers that were included in the IRDTs were used for the evaluations. The average minimum virus titer required for a positive reaction in two examinations is shown in the tables.

**TABLE 1:** Influenza rapid diagnosis tests (IRDTs) evaluated in this study.

**IRDT**	**Manufacturer**	**Country of origin of IRDT**	**Format^a^**	**Input ratio^b^ (%)**	**Minutes to assess^c^**
Statmark FLU Stick-N	Nichirei Biosciences	Japan	Test strip	100	1–5
RapidTesta colorFLU stick	Sekisui Medical	United States	Test strip	100	2–10
QuickVue Rapid SP influ	DS Pharma Biomedical	United States	Test strip	100	10
Clearview Exact Influenza A&B	Alere Medical	United States	Test strip	100	8
Espline Influenza A&B-N	Fujirebio	Japan	Well	6.7	15
ImmunoAce Flu	Tauns Laboratories	Japan	Well	12.5	3–5
Brightpoc Flu	Nichirei Biosciences	Japan	Well	13.8	1–10
Immunofine FLU	Nichirei Biosciences	Japan	Well	13.8	1–5
QuickNavi Flu	Denka Seiken	Japan	Well	10	8
QuickNavi-Flu + RSV	Denka Seiken	Japan	Well	10	8
Prorast Flu One	Adtec	Japan	Well	16.7	8
Finevision Influenza	Alere Medical	United States	Well	10	5
Nanotrap Flu A⋅B	Rohto Pharmaceutical	Japan	Well	8.3	3–8
Quick Chaser Flu A, B (Type H)	Mizuho Medy	Japan	Well	25	5–10
Alsonic Flu	Alfresa pharma	Japan	Well	10	5
Primecheck Flu (Type S)	Alfresa pharma	Japan	Well	20.8	3–10
RapidTesta FLU⋅NEXT	Sekisui Medical	United States	Well	24	15
Spotchem i-Line FluAB-S	Arkray-factory	Japan	Well	8.75	1–10
QuickNavi Flu2	Denka Seiken	Japan	Well + Analyzer	10	5
BD Veritor System Flu	Becton Dickinson	United States	Well + Analyzer	13.3	5–10
Fuji dri-chem immuno AG cartridge FluAB	Mizuho Medy	Japan	Well + Analyzer	21.4	3–10
Spotchem FLORA FluAB	Arkray-factory	Japan	Well + Analyzer	13.6	1.5–10
Sofia Influenza A + B FIA	DS Pharma Biomedical	United States	Well + Analyzer	30	15

*^*a*^All IRDTs examined were divided into two types based on their format: (i) test strip format, in which a test strip is dipped into the lysed sample and the reaction occurs on the strip; or (ii) well format, in which the lysed sample is dropped into the well and the reaction occurs inside a covered plastic body. “+ Analyzer” means that these IRDTs need an analyzer to evaluate the result. ^*b*^For all tested IRDTs, the test samples (100 μl) were mixed with lysis buffer (A). All or part of the lysed sample (B) was subjected to the assay. Input ratios were calculated using the following formula: volume B/(100 μl + volume A) × 100. ^*c*^The time required to obtain the results is based on the individual manufacturer’s instructions.*

### Viruses

The influenza viruses listed in Table 2 were propagated in MDCK cells, AX4 cells, which express higher amounts of 6-linked sialic acids compared with MDCK cells on their cell surface via exogenous expression of human β-galactoside α2,6-sialyltransferase I (ST6Gal) (Hatakeyama et al., 2005), or chicken embryonated eggs (Table 2). Virus titers (TCID_50_) were determined by using MDCK cells. Test samples (100 μl) were adjusted to 10^1^–10^6^ TCID_50_ with Eagle’s minimal essential medium containing 0.3% bovine serum albumin.

**TABLE 2:** Influenza virus isolates used in this study.

**Classification**	**Virus strain**	**Abbreviation^b^**	**Propagated in**
Seasonal H1N1pdm09	A/Tokyo/UT-HP013/2016	H1-1	MDCK cells
	A/Tokyo/UT-BB131/2017	H1-2	MDCK cells
	A/Tokyo/UT001-1/2018	H1-3	MDCK cells
Seasonal H3N2	A/Tokyo/UT-HP022/2016	H3-1	MDCK cells
	A/Tokyo/UT-HP036/2017	H3-2	AX4 cells
	A/Tokyo/UT002-1/2018	H3-3	MDCK cells
Seasonal B/Victoria	B/Tokyo/UT-HP009/2016	Vic-1	MDCK cells
	B/Tokyo/UT-DA25-2/2017	Vic-2	MDCK cells
	B/Tokyo/UT-WD028/2018	Vic-3	MDCK cells
Seasonal B/Yamagata	B/Tokyo/UT-HP005/2016	Yam-1	MDCK cells
	B/Tokyo/UT-HP053/2017	Yam-2	MDCK cells
	B/Tokyo/UT-HP073/2018	Yam-3	MDCK cells
H5 viruses	A/chicken/Japan/AQ-HE79/2015 (H5N1)^a^	H5-1	Embryonated chicken eggs
	A/duck/Japan/AQ-HE29-79/2017 (H5N1)^a^	H5-2	Embryonated chicken eggs
	A/muscovy duck/Aomori/1-3T/2016 (H5N6)^a^	H5-3	Embryonated chicken eggs
	A/chicken/Niigata/1-1T/2016 (H5N6)^a^	H5-4	Embryonated chicken eggs
H7 viruses	A/chicken/Kagawa/1T/2018 (H5N6)^a^	H5-5	Embryonated chicken eggs
	A/Anhui/1/2013 (H7N9)	H7-1	Embryonated chicken eggs
	A/Guangdong/17SF003/2016 (H7N9)^a^	H7-2	Embryonated chicken eggs
	A/duck/Japan/AQ-HE29-22/2017 (H7N9)^a^	H7-3	Embryonated chicken eggs

*^*a*^Viruses possess HA with multi-basic amino acids at the cleavage site. ^*b*^Abbreviations used in Tables 3, 4 are shown.*

### Biosafety Statements

All experiments with H5N1, H5N6, and H7N9 viruses were performed in biosafety level 3 (BSL3) laboratories at the University of Tokyo, which are approved for such use by the Ministry of Agriculture, Forestry and Fisheries, Japan.

## Results and Discussion

We examined the sensitivity of 23 IRDTs commercially available in the 2018–2019 influenza season (Table 1). All of the IRDTs tested employed an immunochromatographic method to detect seasonal influenza A and B viruses. Therefore, the sensitivity of the IRDTs was primarily determined by the binding kinetics of the monoclonal antibody used in the IRDT. RapidTesta FLU⋅NEXT uses a rat monoclonal antibody against the nucleoprotein (NP) of influenza A virus, whereas the others use mouse monoclonal antibodies against the NPs of influenza A and B viruses. Because the epitopes of these monoclonal antibodies on the NP of influenza A virus are conserved among influenza A viruses, 19 of the 23 IRDTs (the exceptions being QuickNavi Flu, QuickNavi-Flu + RSV, Nanotrap Flu A⋅B, and QuickNavi Flu2) claim to be able to detect several animal influenza A viruses, including subtypes H1 through H16, in their appendixes. Although amino acid substitutions in NP are found less frequently than those in HA, NP mutations do occur in seasonal influenza viruses and may affect the sensitivity of IRDTs.

The composition of the lysis buffer, the proportion of sample in the analyte, and the method used to visualize the result also affect the sensitivity. The proportion of sample in the analyte is heavily determined by the format of the IRDT; that is, whether it has the test strip format or the well format (Format column, Table 1). The entire test sample can be utilized with the test strip format, whereas the input ratio of the test samples is limited to between 6.7 and 30% for the well format (Input ratio column, Table 1). The method for visualization of the results also differs among the 23 IRDTs: Espline Influenza A&B-N uses alkaline phosphatase and its substrate, Spotchem FLORA FluAB uses a fluorescent latex microsphere, Sofia Influenza A + B FIA uses europium, and the others use gold-, gold plus silver-, gold plus platinum-, or latex-non-fluorescent microspheres for visualization of the immune-complex. The visualized results are assessed by a specific analyzer for QuickNavi Flu2, BD Veritor System Flu, Fuji dri-chem immuno AG cartridge FluAB, Spotchem FLORA FluAB, and Sofia Influenza A + B FIA; the human eye is used for the others (Table 1). All 23 IRDTs requires 1–15 min to complete each test.

We examined the sensitivity of these 23 IRDTs for each of three isolates of H1N1pdm09, H3N2, B/Victoria-lineage, and B/Yamagata-lineage isolated between 2016 and 2018 (Table 2). The detection limit of the IRDTs for seasonal H1N1pdm09 and H3N2 viruses ranged from 10^1^ to 10^6^ TCID_50_ per 100 μl (Table 3). The sensitivity for H3N2 viruses tended to be lower than that for H1N1pdm09 viruses. A similar trend was observed in our previous reports (Sakai-Tagawa et al., 2014, 2017). The detection limit of the IRDTs tested for the B/Victoria- and B/Yamagata-lineages ranged from 10^2^ to 10^6^ TCID_50_ per 100 μl (Table 3). All tested IRDTs possessed similar sensitivity for B/Victoria- and B/Yamagata-lineages but lower or similar sensitivity for seasonal influenza B viruses compared with that for seasonal influenza A viruses (Table 3). The most sensitive IRDT for seasonal influenza A and B viruses was Sofia Influenza A + B FIA.

**TABLE 3:** Sensitivity of IRDTs for seasonal influenza A and B viruses^a^.

**IRDT**	**Minimum virus titer (log_10_ TCID_50_/100 μl) for a positive result with**											
	**H1-1**	**H1-2**	**H1-3**	**H3-1**	**H3-2**	**H3-3**	**Vic-1**	**Vic-2**	**Vic-3**	**Yam-1**	**Yam-2**	**Yam-3**
Statmark FLU Stick-N	4	5	3	5	6	5	5	4	4	5	5	4
RapidTesta colorFLU stick	4	4	3	5	6	5	5	4	4	4	4	3
QuickVue Rapid SP influ	4	5	3	5	5	5	5	4	4	4	4	3
Clearview Exact Influenza A&B	4	4	3	5	6	5	4	3	3	3	3	3
Espline Influenza A&B-N	4	4	2	4	5	4	4	3	4	4	3.5	3
ImmunoAce Flu	4	4	2	5	6	4.5	4.5	3.5	4	4	4	3
Brightpoc Flu	4	4	3	5	6	5	5	4	4	4	4	4
Immunofine FLU	4	4	3	5	6	5	5	4	4.5	4	4	3
QuickNavi Flu	4	4	3	5	6	5	6	5	5	5	5	5
QuickNavi-Flu + RSV	4	4	3	5	6	5	6	5	5	5	5	5
Prorast Flu One	3	4	2	4	5	4	5	4	5	5	5	4
Finevision Influenza	4	4	3	5	5	4	5	4	4	4	4	3
Nanotrap Flu A⋅B	4	4	3	5	6	5	5	4	4	4	4	4
Quick Chaser Flu A,B (Type H)	4	4	2	5	5	4	4	3	4	4	4	3
Alsonic Flu	4	4	3	5	6	5	5	4	4	4	4	3
Primecheck Flu (Type S)	4	4	3	4	5	4	5	4	4	4	4	3
RapidTesta FLU⋅NEXT	3	3	2	4	5	4	4	3	3.5	4	4	3
Spotchem i-Line FluAB-S	4	4	3	5	6	5	5.5	5	5	6	5	5
QuickNavi Flu2	4	4	3	5	5	4.5	5.5	5	5	5	5	5
BD Veritor System Flu	4	4	2	5	5	4	4	3	3	4	4	3
Fuji dri-chem immuno AG cartridge FluAB	3	3	1.5	3.5	4.5	3	4	3	3.5	3	3	3
Spotchem FLORA FluAB	3	3	2	4	5	4	4	3	3	3.5	3	2.5
Sofia Influenza A + B FIA	2	2	1	3	4	3	4	2.5	3	3	3	2

*^*a*^Ten-fold serial dilutions of the indicated viruses (10^1^–10^6^ TCID_50_ per 100 μl) were examined with each IRDT according to the manufacturers’ instructions. The minimum viral titers required for a positive reaction were determined in two independent experiments. The average titers are shown.*

We next tested the sensitivity of the 23 IRDTs against H5N1 and H5N6 viruses. The two H5N1 viruses (H5-1 and H5-2) were isolated from raw poultry products that were illegally brought into Japan by international flight passengers (Shibata et al., 2018a) and the three H5N6 viruses (H5-3, H5-4, and H5-5) were isolated from bird feces in Japan (Uchida et al., 2019). These H5 viruses are circulating in avian species and have the potential to transmit to humans (Nunez and Ross, 2019). The detection limit of the 23 IRDTs ranged from 10^3^ to 10^5^ TCID_50_ per 100 μl for the two H5N1 viruses (H5-1 and H5-2) and from 10^2^ to 10^6^ TCID_50_ per 100 μl for the three H5N6 viruses (H5-3, H5-4, and H5-5) (Table 4). The sensitivity of the IRDTs for H5-3 and H5-4 was 10–100 lower than that for H5-5. For H7N9 viruses, we used a prototype H7N9 virus (H7-1) that was isolated from the first reported human H7N9 case in 2013 (Gao et al., 2013) and a highly pathogenic H7N9 isolate (H7-2) that emerged and was isolated from a human in 2017 (Zhang et al., 2017; Zhu et al., 2017). Another highly pathogenic H7N9 isolate (H7-3) was isolated in 2017 from Muscovy duck meat, which was brought by passengers from China to Japan (Shibata et al., 2018b). The detection limits of the 23 IRDTs for these H7N9 viruses ranged from 10^1^ to 10^6^ TCID_50_ per 100 μl (Table 4). The sensitivity of the IRDTs for H7-3 was 100–1000 higher than that for H7-1 and H7-2. Several IRDTs detected the H5 and H7 viruses and the seasonal viruses with similar sensitivity. The most sensitive IRDT for H5Nx and H7N9 viruses was Sofia Influenza A + B FIA.

**TABLE 4:** Sensitivity of IRDTs for H5 and H7 viruses^a^.

**IRDT**	**Minimum virus titer (log_10_ TCID_50_/100 μl) for a positive result with**							
	**H5-1**	**H5-2**	**H5-3**	**H5-4**	**H5-5**	**H7-1**	**H7-2**	**H7-3**
Statmark FLU Stick-N	5	5	6	6	4	5	6	3.5
RapidTesta colorFLU stick	5	5	6	6	4	5	5	3
QuickVue Rapid SP influ	5	4	6	6	4	5	5	3
Clearview Exact Influenza A&B	5	4	6	5.5	4	4	5	3
Espline Influenza A&B-N	5	4	5	5	4	4	5	3
ImmunoAce Flu	5	4	6	6	4	4.5	5	3
Brightpoc Flu	5	4	6	6	4	5	5	3
Immunofine FLU	5	4	6	6	4	5	5	3
QuickNavi Flu	4	4	5	5	4	4	5	3
QuickNavi-Flu + RSV	4.5	4	5	5	4	4.5	5	3
Prorast Flu One	4	4	5	5	4	4	5	3
Finevision Influenza	5	5	6	6	4	5	5	3
Nanotrap Flu A⋅B	5	4	6	6	4	5	5	3
Quick Chaser Flu A,B (Type H)	5	4	6	6	4	4	5	2.5
Alsonic Flu	5	4	6	6	4	4.5	5	3
Primecheck Flu (Type S)	5	4	5	5	4	4	5	2
RapidTesta FLU⋅NEXT	4	4	5	5	3	4	5	2
Spotchem i-Line FluAB-S	5	5	6	6	4	5	6	3.5
QuickNavi Flu2	4	4	5	5	4	4	5	3
BD Veritor System Flu	5	4	5	5	3	4	5	2
Fuji dri-chem immuno AG cartridge FluAB	4	3	5	5	2.5	3.5	4	1.5
Spotchem FLORA FluAB	4.5	4	5.5	5	3	4	5	2
Sofia Influenza A + B FIA	4	3	4	4	2	3	4	1

*^*a*^Ten-fold serial dilutions of the indicated viruses (10^1^–10^6^ TCID_50_ per 100 μl) were examined with each IRDT according to the manufacturers’ instructions. The minimum viral titers required for a positive reaction were determined in two independent experiments. The average titers are shown.*

In this study, all tested IRDTs possessed sufficient sensitivity to detect seasonal H1N1pdm09, H3N2, and type B viruses, as well as H5N1, H5N6, and H7N9 viruses, which are potentially transmittable to humans (Imai et al., 2017; Nunez and Ross, 2019). Most of the IRDTs tested in this study showed similar sensitivity for seasonal influenza A and B viruses as the IRDTs we tested previously (Sakai-Tagawa et al., 2017). Sofia Influenza A + B FIA showed the best sensitivity to all of the influenza A and B viruses tested. Of note, the sensitivity of several of the IRDTs for seasonal influenza viruses differed by 10 times compared with our previous study (Sakai-Tagawa et al., 2017). This difference may be due to modifications of the IRDTs to improve their sensitivity to recent seasonal influenza viruses. Therefore, we need to continue to periodically monitor the sensitivity of IRDTs.

The sensitivity of the analyzer-based IRDTs was similar to or better than that of the IRDTs assessed by the human eye. In the case of seasonal viruses, virus titers usually reach 10^2^–10^6^ TCID_50_ per 100 μl of nasopharyngeal wash during the first 24–72 h of illness (World Health Organization Writing Group et al., 2006). Therefore, all of the IRDTs tested here would, in theory, detect these influenza viruses in patients during this period. However, to detect influenza viruses within 24 h of onset, more sensitive IRDTs are required. Sofia Influenza A + B FIA, Spotchem FLORA FluAB, and Fuji dri-chem immuno AG cartridge FluAB may be suitable for such use because of their higher sensitivity compared with the others. We also need novel rapid detection kits that can detect NA- or PA-inhibitor resistant viruses to help physicians select the most appropriate antiviral therapy. Sofia Influenza A + B FIA detected the H5 and H7 viruses at a high sensitivity, identical to that for seasonal influenza A and B viruses; the detection limit was 10^1^–10^4^ TCID_50_ per 100 μl, suggesting that this IRDT can diagnose influenza caused by such viruses.

## Data Availability Statement

The raw data supporting the conclusions of this manuscript will be made available by the authors, without undue reservation, to any qualified researcher.

## Author Contributions

YK designed the study. YS-T performed the experiments. YS-T and SY analyzed the data. SY and YK wrote the manuscript. All authors reviewed and approved the manuscript.

## Conflict of Interest

YK has received speaker’s honoraria from Toyama Chemical and Astellas Inc.; has received grant support from Chugai Pharmaceuticals, Daiichi Sankyo Pharmaceutical, Toyama Chemical, Tauns Laboratories, Inc., Tsumura & Co., and Denka Seiken Co., Ltd.; and is a co-founder of FluGen. The remaining authors declare that the research was conducted in the absence of any commercial or financial relationships that could be construed as a potential conflict of interest.
